# Supplementary material for: Unveiling the Bioactive Potential of the Invasive Jellyfish Phyllorhiza punctata Through Integrative Transcriptomic and Proteomic Analyses
Source: Biomolecules. 2025 Aug 4;15(8):1121. doi: 10.3390/biom15081121 (PMC12383608; doi:10.3390/biom15081121)
Supplement: Supplementary file 1 [file biomolecules-15-01121-s001.zip › Suplementary Material/FileS3/PEPFOLD4/QKN61214_16427/clusters.html]

 PEP-FOLD clusters


Download archive of all models  here   

|  |  |  |
| --- | --- | --- |
| # | file | sOPEP |
| # cluster 1 - Size: 1 |  |  |
|  | QKN61214\_16427-model1 | -22.1928 |
| # cluster 2 - Size: 5 |  |  |
|  | QKN61214\_16427-model2 | -17.5117 |
|  | QKN61214\_16427-model2.1 | -17.4308 |
|  | QKN61214\_16427-model2.2 | -17.1946 |
|  | QKN61214\_16427-model2.3 | -17.111 |
|  | QKN61214\_16427-model2.4 | -16.9333 |
| # cluster 3 - Size: 63 |  |  |
|  | QKN61214\_16427-model3 | -17.3572 |
|  | QKN61214\_16427-model3.1 | -17.3535 |
|  | QKN61214\_16427-model3.2 | -17.317 |
|  | QKN61214\_16427-model3.3 | -17.313 |
|  | QKN61214\_16427-model3.4 | -17.313 |
|  | QKN61214\_16427-model3.5 | -17.3128 |
|  | QKN61214\_16427-model3.6 | -17.3114 |
|  | QKN61214\_16427-model3.7 | -17.3108 |
|  | QKN61214\_16427-model3.8 | -17.2794 |
|  | QKN61214\_16427-model3.9 | -17.2794 |
|  | QKN61214\_16427-model3.10 | -17.2794 |
|  | QKN61214\_16427-model3.11 | -17.2722 |
|  | QKN61214\_16427-model3.12 | -17.2509 |
|  | QKN61214\_16427-model3.13 | -17.2428 |
|  | QKN61214\_16427-model3.14 | -17.2417 |
|  | QKN61214\_16427-model3.15 | -17.2341 |
|  | QKN61214\_16427-model3.16 | -17.2286 |
|  | QKN61214\_16427-model3.17 | -17.2255 |
|  | QKN61214\_16427-model3.18 | -17.2121 |
|  | QKN61214\_16427-model3.19 | -17.2116 |
|  | QKN61214\_16427-model3.20 | -17.2069 |
|  | QKN61214\_16427-model3.21 | -17.1939 |
|  | QKN61214\_16427-model3.22 | -17.1889 |
|  | QKN61214\_16427-model3.23 | -17.1801 |
|  | QKN61214\_16427-model3.24 | -17.1793 |
|  | QKN61214\_16427-model3.25 | -17.173 |
|  | QKN61214\_16427-model3.26 | -17.1719 |
|  | QKN61214\_16427-model3.27 | -17.1714 |
|  | QKN61214\_16427-model3.28 | -17.1569 |
|  | QKN61214\_16427-model3.29 | -17.1505 |
|  | QKN61214\_16427-model3.30 | -17.1505 |
|  | QKN61214\_16427-model3.31 | -17.1493 |
|  | QKN61214\_16427-model3.32 | -17.1493 |
|  | QKN61214\_16427-model3.33 | -17.1368 |
|  | QKN61214\_16427-model3.34 | -17.1331 |
|  | QKN61214\_16427-model3.35 | -17.1156 |
|  | QKN61214\_16427-model3.36 | -17.1096 |
|  | QKN61214\_16427-model3.37 | -17.1084 |
|  | QKN61214\_16427-model3.38 | -17.0997 |
|  | QKN61214\_16427-model3.39 | -17.0901 |
|  | QKN61214\_16427-model3.40 | -17.088 |
|  | QKN61214\_16427-model3.41 | -17.0841 |
|  | QKN61214\_16427-model3.42 | -17.0789 |
|  | QKN61214\_16427-model3.43 | -17.0789 |
|  | QKN61214\_16427-model3.44 | -17.0737 |
|  | QKN61214\_16427-model3.45 | -17.0666 |
|  | QKN61214\_16427-model3.46 | -17.048 |
|  | QKN61214\_16427-model3.47 | -17.0392 |
|  | QKN61214\_16427-model3.48 | -17.033 |
|  | QKN61214\_16427-model3.49 | -17.0319 |
|  | QKN61214\_16427-model3.50 | -17.0313 |
|  | QKN61214\_16427-model3.51 | -17.0286 |
|  | QKN61214\_16427-model3.52 | -17.016 |
|  | QKN61214\_16427-model3.53 | -17.016 |
|  | QKN61214\_16427-model3.54 | -17.0125 |
|  | QKN61214\_16427-model3.55 | -16.9969 |
|  | QKN61214\_16427-model3.56 | -16.9903 |
|  | QKN61214\_16427-model3.57 | -16.9885 |
|  | QKN61214\_16427-model3.58 | -16.9813 |
|  | QKN61214\_16427-model3.59 | -16.9696 |
|  | QKN61214\_16427-model3.60 | -16.9057 |
|  | QKN61214\_16427-model3.61 | -16.889 |
|  | QKN61214\_16427-model3.62 | -16.8828 |
| # cluster 4 - Size: 10 |  |  |
|  | QKN61214\_16427-model4 | -17.3325 |
|  | QKN61214\_16427-model4.1 | -17.1648 |
|  | QKN61214\_16427-model4.2 | -17.1063 |
|  | QKN61214\_16427-model4.3 | -17.0977 |
|  | QKN61214\_16427-model4.4 | -17.0661 |
|  | QKN61214\_16427-model4.5 | -17.0398 |
|  | QKN61214\_16427-model4.6 | -16.9987 |
|  | QKN61214\_16427-model4.7 | -16.9871 |
|  | QKN61214\_16427-model4.8 | -16.9685 |
|  | QKN61214\_16427-model4.9 | -16.6851 |
| # cluster 5 - Size: 8 |  |  |
|  | QKN61214\_16427-model5 | -17.2368 |
|  | QKN61214\_16427-model5.1 | -17.1928 |
|  | QKN61214\_16427-model5.2 | -17.1886 |
|  | QKN61214\_16427-model5.3 | -17.1297 |
|  | QKN61214\_16427-model5.4 | -17.0123 |
|  | QKN61214\_16427-model5.5 | -16.9976 |
|  | QKN61214\_16427-model5.6 | -16.9721 |
|  | QKN61214\_16427-model5.7 | -16.9656 |
| # cluster 6 - Size: 2 |  |  |
|  | QKN61214\_16427-model6 | -17.1884 |
|  | QKN61214\_16427-model6.1 | -17.0895 |
| # cluster 7 - Size: 2 |  |  |
|  | QKN61214\_16427-model7 | -17.0629 |
|  | QKN61214\_16427-model7.1 | -17.0086 |
| # cluster 8 - Size: 2 |  |  |
|  | QKN61214\_16427-model8 | -14.314 |
|  | QKN61214\_16427-model8.1 | -13.9655 |
| # cluster 9 - Size: 1 |  |  |
|  | QKN61214\_16427-model9 | -13.8024 |
| # cluster 10 - Size: 2 |  |  |
|  | QKN61214\_16427-model10 | -13.7163 |
|  | QKN61214\_16427-model10.1 | -13.6149 |
| # cluster 11 - Size: 2 |  |  |
|  | QKN61214\_16427-model11 | -13.6516 |
|  | QKN61214\_16427-model11.1 | -13.4511 |
| # cluster 12 - Size: 1 |  |  |
|  | QKN61214\_16427-model12 | -12.2244 |
| # cluster 13 - Size: 1 |  |  |
|  | QKN61214\_16427-model13 | -7.69421 |
|  |  |  |
